# Parsimonious Random-Forest-Based Land-Use Regression Model Using Particulate Matter Sensors in Berlin, Germany

**DOI:** 10.3390/s24134193

**Published:** 2024-06-27

**Authors:** Janani Venkatraman Jagatha, Christoph Schneider, Tobias Sauter

**Affiliations:** Geography Department, Humboldt-Universität zu Berlin, Unter den Linden 6, 10099 Berlin, Germany; christoph.schneider@geo.hu-berlin.de (C.S.); tobias.sauter@geo.hu-berlin.de (T.S.)

**Keywords:** particulate matter, sensitivity analysis, random forest, regression modelling, land use

## Abstract

Machine learning (ML) methods are widely used in particulate matter prediction modelling, especially through use of air quality sensor data. Despite their advantages, these methods’ black-box nature obscures the understanding of how a prediction has been made. Major issues with these types of models include the data quality and computational intensity. In this study, we employed feature selection methods using recursive feature elimination and global sensitivity analysis for a random-forest (RF)-based land-use regression model developed for the city of Berlin, Germany. Land-use-based predictors, including local climate zones, leaf area index, daily traffic volume, population density, building types, building heights, and street types were used to create a baseline RF model. Five additional models, three using recursive feature elimination method and two using a Sobol-based global sensitivity analysis (GSA), were implemented, and their performance was compared against that of the baseline RF model. The predictors that had a large effect on the prediction as determined using both the methods are discussed. Through feature elimination, the number of predictors were reduced from 220 in the baseline model to eight in the parsimonious models without sacrificing model performance. The model metrics were compared, which showed that the parsimonious_GSA-based model performs better than does the baseline model and reduces the mean absolute error (MAE) from 8.69 µg/m^3^ to 3.6 µg/m^3^ and the root mean squared error (RMSE) from 9.86 µg/m^3^ to 4.23 µg/m^3^ when applying the trained model to reference station data. The better performance of the GSA_parsimonious model is made possible by the curtailment of the uncertainties propagated through the model via the reduction of multicollinear and redundant predictors. The parsimonious model validated against reference stations was able to predict the PM_2.5_ concentrations with an MAE of less than 5 µg/m^3^ for 10 out of 12 locations. The GSA_parsimonious performed best in all model metrics and improved the R^2^ from 3% in the baseline model to 17%. However, the predictions exhibited a degree of uncertainty, making it unreliable for regional scale modelling. The GSA_parsimonious model can nevertheless be adapted to local scales to highlight the land-use parameters that are indicative of PM_2.5_ concentrations in Berlin. Overall, population density, leaf area index, and traffic volume are the major predictors of PM_2.5_, while building type and local climate zones are the less significant predictors. Feature selection based on sensitivity analysis has a large impact on the model performance. Optimising models through sensitivity analysis can enhance the interpretability of the model dynamics and potentially reduce computational costs and time when modelling is performed for larger areas.

## 1. Introduction

Around 10% of the people living in Berlin, Germany, reside in areas with a very low or low development index [1], which is directly associated with a higher risk of exposure to particulate matter (PM) and other air pollutants [2]. Located at the mid-latitude ranges in the Northern European plateau, Berlin is the largest German city and the most populous city in the European Union [3]; thus, the local population is subject to significantly increasing health risks due to poor air quality. Berlin’s climate is dominated by a modified maritime air mass originating from the Atlantic from a southwest to northwest wind direction [4]. Regularly occurring weather episodes with air flow from the east are typically associated with lower wind speed and overall elevated levels of PM concentrations [5]. In the last decade, the annual mean concentration of PM_2.5_ in Germany has reduced from approximately 20 µg/m^3^ to approximately 7 µg/m^3^. However, the World Health Organisation’s recommended limit value of 5 µg/m^3^ (annual mean) is exceeded in 99.5% of the stations in Germany [6]. Globally, within urban areas, the sources of PM_2.5_ are a mix of local sources such as traffic, cooking, construction activities, power generation [7], formation of secondary particles [8], and long-range transport of dust [9]. PM concentrations in Berlin are attributed to both long-range transport and local sources [10,11]. In general, the largest contribution to the PM_2.5_ concentration in Berlin is from locally produced sources such as traffic-related emissions, households, and industry, followed by long-range transportation of PM from other German cities and transboundary sources [11]. Knowledge of the micro-scale variability of PM, and thus the quantification of the exposure risk, could significantly reduce health risks and increase the quality of life and well-being of citizens [12,13]. This can be achieved through city-planning approaches that are informed by the knowledge of the relationship between urban structures and PM concentration [14,15]. In this study, we aimed to develop a prediction model for PM_2.5_ that can be used at the local and regional scales. We combined the advantages of machine learning (ML) and sensitivity analysis to develop a parsimonious random-forest-based land-use regression model.

Modelling of air pollution is carried out through land-use-based models [15,16,17], Bayesian network probability models [18], multi-objective scheduling models [19], satellite-based models using aerosol optical depth [20,21], meteorology-based models with regression and ML [22,23], and urban-scale models [24,25]. Land-use-based regression models (LURMs) are commonly used to understand how urban design, land use, and socioeconomic factors [15,16,17,26] interact with air pollution. LURMs are especially useful in locations where air pollution data are unavailable [27]. The spatial distributions can be derived by correlating air pollution with land-used-based predictors [28,29], local sources of emissions [28,29], and meteorology [30]. The review articles by Azmi et al. (2023) [16], Hoek et al. (2008) [15], and Ryan et al. (2007) [17] provide a comprehensive overview of LURM studies for air pollution parameters such as volatile organic compounds, nitrogen oxides, ozone, and PM. The coefficient of determination (R^2^) is used in most of the studies to compare the models and evaluate the model performance. Mean error and cross-validation techniques are also used as model evaluation metrics in studies that have employed ML methods to develop LURMs [31,32,33].

The applicability of LURMs beyond the extent of the official measurement networks has been the focus of many studies. For example, the study by Merbitz et al. (2012) [29] aimed to create a statistical model for PM_10_ and PM_2.5_ using a small number of predictors, including the traffic-emissions-based concentration profile (relative concentration to the source as a function of distance) and land-use classification simplified to two categories—building density (all building classes grouped together) and green area (urban green and forest). They did not discriminate between residential and commercial buildings. Although all three predictors correlated with PM, it was concluded that the results were better for PM_10_ than for PM_2.5_, as PM_10_ is more directly influenced by local sources than is PM_2.5_. The model tended to largely underestimate concentrations in open areas, temporary traffic hotspots, and street canyons. Hoogh et al. (2016) [34] developed a LURM for PM_2.5_ and nitrogen dioxide data incorporating satellite and chemical transport modelling data. They used data from the European AIRBASE network and the European Study of Cohorts for Air Pollution Effects measurement sites air quality database as ground-based data sets. They used 80% of the monitoring sites to train and 20% to test and validate and achieved an R^2^ of 0.6. However the AIRBASE data set is now depreciated. The study by Ge et al. (2023) [32] used the two ML models, least absolute selection and a shrinkage-operator (LASSO)-enhanced random forest (RF) model called LASSO-RERF to extrapolate PM_2.5_ data from the regulatory monitoring network and low-cost sensor network to sparsely monitored areas. Using this method, Ge et al. (2023) [32] could improve the R^2^ from 0.49 to 0.65, and the RMSE from 3.56 µg/m^3^ to 2.96 µg/m^3^. Kumar et al. (2020) [35] also combined two ML models, extra-trees regression and Ada-Boost to develop a LURM using meteorological variables to estimate PM_2.5_ concentrations in Delhi, India.

Due to microscale variations in PM concentrations, it is a challenge to develop LURMs that are transferable to other areas [15]. For example, the LURM developed for 20 European cities by Eeftens et al. (2012) [31] could not be generalised for all cities and had to be locally optimised for each study area. The simplicity of air quality sensors (AQSs) and advantages due to their portability and longer battery life, helps in capturing the microscale variation in PM concentrations in more detail [36]. These kinds of studies are not feasible with the use of conventional stationary measurements using reference-grade devices due to the higher costs involved to procure and safeguard the devices [37]. The usage of sensors to measure air quality has spread rapidly in the last decade due to the advances in micro-sensor technology, modern production facilities, and reduced development costs [38,39]. In contrast to reference-standard devices, AQSs are simple in design, lightweight, and easier to deploy in large numbers [40,41]. AQSs are also used in mixed networks in combination with satellite images and/or monitoring stations [42,43]. AQSs perform well under laboratory and stationary conditions but have a low temporal resolution of 30 to 60 min [44,45]. AQSs on mobile platforms need to operate with a higher temporal resolution in order to compensate for the vehicle speed and capture as many data points as possible. This leads to mobile AQSs needing to have higher uncertainties due to road conditions, vibrations, and wind [46]. Nevertheless, with the good measurement practises as outlined in [39], AQSs offer the capability to generate high volumes of data at reasonably low cost. A LURM based on data collected from mobile sampling could potentially offer a highly cost-effective approach to modelling and mapping air pollution concentration levels in spatially continuous ways [47].

ML methods are widely used in PM prediction models using air quality data from officially run stations [48], AQSs [30,49], satellite images [21], meteorological data such as temperature and precipitation [50], and urban structures [30]. Several ML methods can be used for prediction of PM depending on the availability, type (images or text), quality, and quantity of data. Furthermore, the type of study, use of a time-series or cross-sectional design, and the relationship between PM and the variables used to predict it play an important role in determining the ML model to be used [51]. Since each ML model has its own strengths and weaknesses, many studies such as those by Murugan et al. (2021) [52], Maaloul et al. (2022) [53], Yazdi et al. (2020) [54], Analitis et al. (2020) [55], Du et al. (2023) [56], and Tian et al. (2021) [57] have used multiple models to compare and contrast the ML models against each other in order to find the model that fits best to the particular study case. The majority of these studies have concluded that random forest (RF) performs best. Tian et al. (2021) [57] concluded that tree-based models (RF and gradient boosting), and neural network models (back propagation neural network and Elman neural network) produce similar estimations but that RF has the best estimation accuracy. Support-vector machine and generalized additive models were also examined but were found to result in worse performance. Similar results were found in studies by Mandal et al. (2020) [58] and Chen et al. (2019) [59]. XGBoost has a good predictive accuracy [60] and can perform better than RF models [61,62]. However, these studies used stationary measurements for training and testing their model.

RF is a nonlinear estimator which fits an ensemble of decision trees on subsets of the data and calculates a mean over all decision trees [63]. By creating multiple subsets, RF improves the predictive accuracy, particularly for nonlinear data [64]. Compared to decision trees, RF is better suited to handling high-dimensional data and preventing overfitting [65]. The RF algorithm is less sensitive to outliers and noisy data as compared to gradient boosting models (GBMs), such as XGBoost and LightGBM, which require the careful tuning of hyper-parameters [66,67]. The RF estimator function in the scikit-learn [68] package introduces randomness into the model through bootstrapping and splitting each node during the construction of a tree. The quality of a split is based on criterion such as mean squared error (MSE), mean absolute error (MAE), and Poisson deviance. The overall variance of the model is therefore reduced by the randomness introduced to the model and by combining individual trees [68].

Despite its advantages, RF estimator is a black-box model. The split criterion of the RF estimator helps in determining the most important predictors or features for the model but does not reduce the number of predictors used. This can affect the quality of the model especially if there are uncertainties in the predictor data, as these uncertainties will be propagated through the model [69]. The second problem with such a model is the multicollinearity of the predictors. Although multicollinearity does not affect the predictive capacity of an RF model, it is a problem when the features need to interpreted. The features determined as important by the model do not necessarily reflect reality, thereby limiting its value for generating interpretations and decisions. The study by Berrocal et al. (2020) [70] compared statistical and ML methods for creating national daily maps for ambient PM_2.5_ concentration in the continental United States and concluded that the numerical methods (such as kriging and statistical downscaling) outperform ML methods, including RF, since they explicitly account for spatial dependence while ML methods do not. The third problem is the computational time and costs of the model: the more predictors used, the more time needed for the model to run through. This especially is exacerbated when the model needs to be run for larger regions.

By removing the inputs that have negligible influence on the output, the model can be simplified and made parsimonious [71]. Recursive feature elimination (RFE) and recursive feature elimination with cross-validation (RFECV) are two types of feature selection methods that are commonly used for reducing the number of predictors for ML models. RFE works by eliminating features by training the model multiple times and then recursively removing the features of low importance until the desired number of features remain [72]. The desired number of features is chosen manually before the model is trained. RFECV, on the other hand, performs RFE in a cross-validation loop to produce the optimal number of features [73]. Despite their effectiveness, these methods do not provide information on the interactions between the predictors. Sensitivity analysis can be an important tool here to understand the dynamic behaviour of the model [74]. Saltelli et al. (2008) [69] define sensitivity analysis as the study of how uncertainty in the output of a model (predicted values) can be apportioned to different sources of uncertainty in the model inputs (predictors). Sensitivity analysis is performed either locally, where the sensitivity of an input parameter is analysed at one at a time [75] or globally, wherein the interactions of an input parameter by itself and with the other input parameters are analysed. Several studies have used sensitivity analysis to enhance their models [76,77,78]. The studies by Todorov et al. (2023) [76] and Wang et al. (2023) [78] are particularly interesting, as they used Sobol-based sensitivity analysis to improve their air quality prediction models. The regional model developed by Todorov et al. (2023) [76] has a resolution of 10 km × 10 km but does not account for PM. Wang et al. (2023) [78] employ global sensitivity analysis (GSA) to an ML model, combining a convolutional neural network and so-called long short-term-memory models to study pollution trends before and during the COVID-19 outbreak.

In this paper, we address predictor selection methods in the context of RF-based LURM for maximum PM_2.5_ (PM_2.5_) prediction in Berlin, Germany. This was achieved by applying RFE, RFECV, and GSA using the Sobol method [69] to a baseline RF model to create a parsimonious RF model with fewer inputs, with validation being conducted via hold-out validation (HOV) [79]. The parsimonious RF model can also be applied to select locations spread across Berlin that correspond to where the regulatory measurement stations run by the Senate Department for Urban Mobility, Transport, Climate Action and the Environment, Berliner Luftgütemessnetz (BLUME), are located [80]. The predicted concentration using the parsimonious RF model was compared against the BLUME station data, which allowed us to examine the possibility of exclusively using AQS data for the LURM. The features of the parsimonious models and their influence on PM_2.5_ concentration in Berlin are discussed since the World Health Organisation’s recommended daily mean concentration of 15 µg/m^3^ (with a maximum of three to four exceedances per year) for PM_2.5_ [81] is exceeded in all of the BLUME stations.

## 2. Methodology

### 2.1. Data Acquisition and Preparation

**Figure 1:**
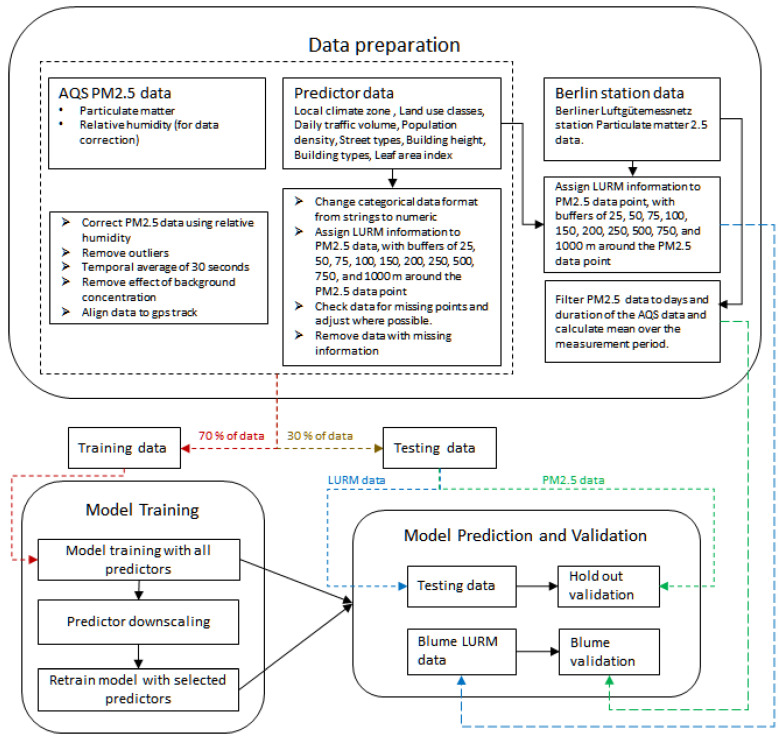
Flowchart showing the methodology of the study.

For the model development, three types of data sets were needed, namely, PM data, land-use data for model development, and PM data for validating the developed model. The acquisition and preparation of each of the three data sets is described in the following sections. Figure 1 shows the methodology for this study.

**Figure 2:**
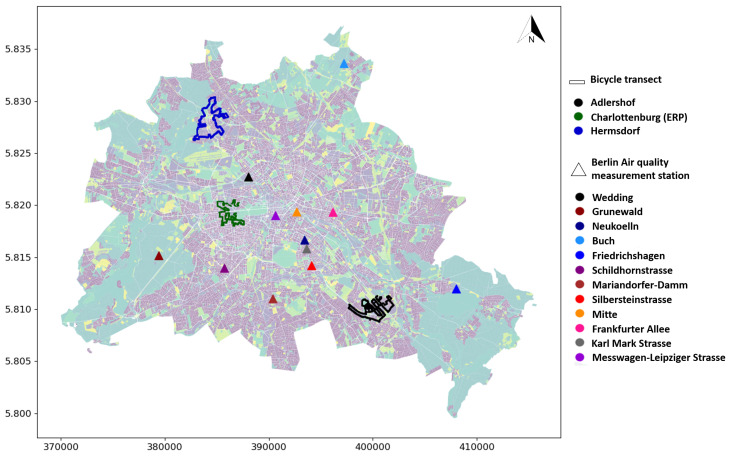
Mobile measurement transects in Berlin-Germany at Hermsdorf, Charlottenburg-Ernst-Reuter-Platz, and Adlershof. The triangular points mark the locations of the official Berlin air quality measurement stations.

**PM Training Data Acquisition:** Three different suburbs in the city of Berlin, Germany—Berlin-Hermsdorf (Hmd), Berlin-Charlottenburg in the vicinity of Ernst Reuter Platz (ERP), and Berlin-Adlershof (Adl) were chosen to collect data for the study (see Figure 2). Berlin-Hermsdorf is located in the northwestern edge of the city bordering Brandenburg. It is characterised by residential areas with a mix of old farm houses, villas and new residential buildings, green areas, and bodies of water. The streets—Hermsdorfer Damm and Heinsestrasse are characterised by higher traffic volumes due to the former’s proximity to the highway A9 and the latter acting as the commercial hub of Hermsdorf [82]. Berlin-Charlottenburg is located in the centre of the city. It is constituted by a mix of residential areas, tall buildings, high commercial activity, wide streets, and large park areas. ERP is a major traffic junction where five major streets—Hardenbergstrasse, Strasse des 17. Juni, Marchstrasse, Otto-Suhr-Allee, and Bismarckstrasse—converge. The Kurfuerstendamm area in Berlin-Charlottenburg is a commercial hub with a large traffic volume and residential area with high population density [83]. Berlin-Adlershof is a suburban area located in the southeastern part of the city. Adlershof consists mostly of large residential areas. The street Rudower Chaussee and the vicinity around it consist mainly of research institutes and university and office buildings. The main federal road, Adlergestell (B96), and highway A113 are prone to high traffic volumes [84].

Bicycle routes covering a distance of approximately 18 km in Hmd, 21 km in ERP, and 27 km in Adl were designed with 11 out of 14 local climate zones (LCZs) that are present in Berlin being taken into account [85]: compact high rise, compact mid-rise, open high rise, open mid-rise, open low rise, large low rise, sparsely built, heavy industry, dense trees, scattered trees, low plants, and water. Three LCZs—sparsely built, bare rock or paved, and bare soil or sand—were not covered within the measurement routes. The measurements in the LCZs of dense trees and water took place at the border of the areas and not in the midst of them. The bicycle routes and their location in Berlin are shown in Figure 2. The details of the bicycle rounds are summarized in Table 1. The measurements were carried out from 15 June 2018, to 15 October 2018, as a part of an intense observation measurement campaign in the Urban Climate Under Change [UC]^2^ project [86]. The measurements at Hmd continued over the winter between 2018 and 2019 until 1 March 2019. A total of 7 rounds in ERP, 15 rounds in Hmd, and 16 rounds in Adl were carried out. However only the measurements taken during summer months—June to October—were considered for this study, limiting the measurements in Hmd to 2 rounds. By limiting the measurements to summer, variations in pollutant levels that would generally occur due to seasonal variations were removed. Additionally, the measurement rounds that were incomplete due to missing PM_2.5_ or GPS data, change in weather conditions (sudden rain), or device malfunction were removed from the analysis.

PM data were collected using an optical particle counter, OPC-N2, from Alphasense, Ltd, Essex, United Kingdom Ltd. [87]. Temperature and relative humidity (RH) were measured using two SHT35 sensors from Sensirion Ltd, Staefa, Switzerland [88]. The sensors, along with the data acquisition system, were built into a metal housing as described in [46]. The sensor ensemble was mounted inside the front basket of a bicycle. The measurements were carried out with a maximum bicycle speed of 15 km/h, with a logging interval for measurement of 2 s. The sensor ensemble was calibrated in the laboratory and in the field [46] using an aerosol spectrometer from Grimm Aerosol Technik Ltd, Ainring, Germany. [89]. The temperature and RH measured are used to account for the meteorological influences on the PM concentration measured. RH is used to correct for hygroscopic growth of PM via Koehler’s theory, as shown in Equations (1) and (2). Equation (1) calculates the correction factor *C* using Koehler’s factor (κ) and particle density (ρ). The correction factor is used to recalculate the *PM* concentrations, with *RH* being taken into account [90,91].

(1) $$C=1+[\frac{\frac{\kappa }{\rho }}{-1+\frac{1}{RH}}]$$

(2) $$PM_{\mathrm{corrected}}=\frac{PM_{\mathrm{uncorrected}}}{C}$$

The measured data of each round are individually handled during pre-processing. First, outliers, here defined as all data in the top and bottom 5th percentile, are removed, and a temporal median of 30 s is calculated. Then, the bottom 5th percentile of the data is assumed to be the background concentration and subtracted from every measurement, and thus only the local concentration of PM is considered for further analysis [92,93]. Additionally, this step eliminates some of the ageing or sensor-drift-induced bias in the data between rounds, with a linear drift being assumed.

The PM_2.5_ data are time and GPS tagged. With the GPS information, an idealised route is created via calculation of the mean of all rounds in a given measurement area. The PM_2.5_ data are then interpolated to the ideal path with all points within a radius of 25 m being considered. The interpolated point contains the information on the maximum, minimum, mean, and standard deviation of PM_2.5_ concentration within a 25 m buffer zone.

**Land-Use Regression Modelling Predictor Data:** The regression model uses spatial predictors for predicting PM concentration. The following predictors are used for the analysis in buffers of 25, 50, 75, 100, 150, 200, 250, 500, 750, and 1000 m radii: local climate zone (LCZ) [85], land-use class (LUC) [94], daily traffic volume (DTV) [95], population density in hectares (PD_ha) [96], OKSTRA (Objektkatalog fuer das Strassen- und Verkehrswesen) street type classification (street_StEP) [97,98,99], RBS (Regionales Bezugssystem) street type classification (street_RBS) [99,100], building height (BH) [101], building type (BT) [101], and leaf area index (LAI) [101].

For each of the predictors, the maximum (max), minimum (min), and mean value for each buffer are calculated. For categorical predictors such as LCZ, LUC, and BT, the most frequently occurring value (cat_max) and the least frequently occurring value (cat_min) are calculated. This is because we use regular grid-point data for categorical variables. In the subsequent analysis, the predictors are named according to the combination of their abbreviation, the statistical means, and buffer size as abbreviation of predictor name_statistic used_buffer-size.

**BLUME Station Data:** The validation of a trained ML model is usually carried out with a subset of the data set used, which is also known as hold-out-method validation (HOV) [79]. However, since this study used AQS data, we checked the possibility of the model to transfer and generalise to the official measurement stations run by the Berlin Senate Department for Urban Mobility, Transport, Climate Action and the Environment, Berliner Luftgütemessnetz (BLUME) [80]. Twelve locations in Berlin that correspond to the official measurement station were chosen for validation. The BLUME stations that collect PM_2.5_ data are classified into three categories: urban background (Wedding, Neukoelln, Mitte), suburban (Grunewald, Buch, Friedrichshagen), and traffic (Messwagen-Leipziger-Strasse, Schildhornstrasse, Mariendorfer Damm, Silbersteinstrasse, Frankfurter Allee, Karl-Marx-Strasse). The LURM predictors within the 10 buffers are assigned to all 12 locations (BLUME_LURM). Similar to the AQS data, the local concentration of PM_2.5_ for each station is calculated by assuming and subtracting the lower 5th percentile as the background concentration. The local maximum is determined by calculating the median over the maximum concentrations of the days when the bicycle measurements took place (BLUME_val).

### 2.2. Model Development

#### 2.2.1. Model Estimator

RF regression is an ensemble technique that makes use of multiple decision trees in determining the final output. Each decision tree is constructed by recursively splitting the training data into subsets based on the values of the model attributes until a criterion is met [63]. RF then performs a bootstrap aggregation, wherein the predictions of all the decision trees are combined in such a way that the overall variance of the model decreases [102,103].

**Figure 3:**
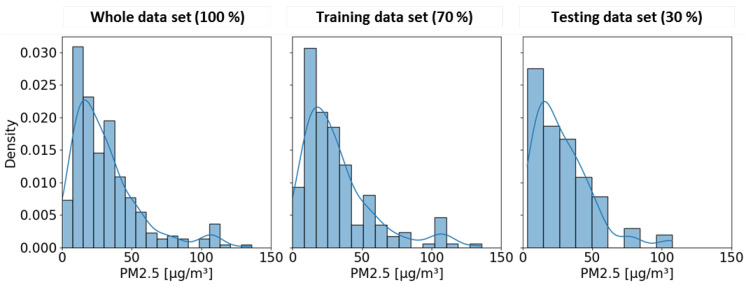
Histogram and density distribution of the PM_2.5_ data used in the study. **Left:** Distribution of the whole data set. **Centre:** Distribution of the training data that constitute 70% of the entire data. **Right:** Distribution of the test data that constitute 30% of the whole data set.

The models were applied using the scikit-learn package [68]. Each variable in the categorical data was numerically coded by assigning a numerical value each to the categorical variable. The points in which any of the predictors had missing data were removed before the analysis. The predictors were not scaled via standardization or normalization, as the RF algorithm is insensitive to it [104]. The data set is split randomly into 70% (584 observations) and 30% (251 observations). The models are trained with the 70% split of the data and tested with the 30% split of the data. The data are split between testing and training in such a way that the distribution is maintained and data from either extremes are included. Figure 3 shows the data distribution for the whole data set (left), the training data (centre), and the testing data (right). The chosen 70% for training covers the entire range covered by the whole data set. The testing data covers all data from 1 µg/m^3^ to 100 µg/m^3^).

#### 2.2.2. Feature Selection

The aim of this study was to develop a parsimonious LURM that is comparable or better to a baseline model consisting of all predictors. Model predictor selection is critical to developing such a parsimonious model. Two methods were used and compared in this study: recursive feature elimination with and without cross-validation and sensitivity analysis using the Sobol method. Both methods work by reducing the variance of the model.

**Recursive feature elimination**: Recursive feature elimination (RFE) is a feature selection method in which the predictors that do not reduce the “impurity” or the chosen criterion such as mean squared error or absolute error of the model are removed. This is done by the iterative training of the model with the chosen estimator—RF in our case—until the desired number of predictors are chosen [68]. RFE can be carried out in two ways: one where the number of predictors are given before training or using cross-validation (RFECV) where the data are divided into multiple subsets consisting of training and testing data sets and where the optimal predictors are derived as a mean of all the models.

In RFE, the model is trained using the test data as a whole. First the model is trained with all the features. The features are then ranked, from 1 to the total number of predictors, based on how much influence they have on the model’s prediction. The least important feature is then eliminated, and the model is trained again until the desired number of features is reached.

RFECV, on the other hand, uses a cross-validation technique wherein the test data are split into *k* subsets. The model is therefore trained on one subset and tested on another subset, thereby training the model *k* − 1 times. By using cross-validation, the model utilises the data efficiently, and resultant model is more reliable [68]. To account for uncertainty, Monte Carlo method [105] is applied, wherein the model is trained 1000 times with a random state parameter of the estimator set from 0 to 1000 (Monte Carlo runs). The features with a mean importance rank of less than 2 are chosen as the important predictors, and the model is retrained and validated.

The three models, RFE, RFECV_baseline (all predictors), and RFECV_parsimonious (RFECV filtered predictors), are assessed and validated. Neither the RFE nor RFECV method provides information on higher-order effects.

**Global sensitivity analysis**: Global sensitivity analysis (GSA) is the process of apportioning the uncertainty of the output to the uncertainty of all the input factors of interest, thereby quantifying the importance of model inputs [76,106,107,108]. This thus allows for the identification of a parameter or a set of parameters that have the largest influence on the model output. For this study, a variance-based sensitivity analysis, also known as the Sobol method [69,109,110], was used to quantify the effect of each of the inputs to the model output variance. We refer to studies such as those by Sauter et al. (2011) [107], Todorov et al. (2023) [76], and Zhang et al. (2015) [77] who describe the principle behind the Sobol method in detail. The Sensitivity Analysis Library (SALib) [111] is used for the analysis.

In order to carry out the GSA, a parameter data set is created using the maximum and minimum value of each predictor. A large enough data set should be created in order to get the best results. The number of simulation runs (*N*) is determined using the desired coefficient of variation (*CV*) of 0.1 and a confidence interval width (*w*) of 0.01, assuming a normal distribution of data from Table 2 of Byrne et al. (2013) [112]. Since the Sensitivity Analysis Library [111] recommends the number of simulations to ideally be a power of 2, *N* is rounded to the next $2^{n}$ number, 2048. The number of data points (*S*) is then calculated using the number of predictors (*D*), as shown in Equation (3).

(3) S=N×(D+2),

In this paper, we assume a uniform distribution for the model inputs. This is because we do not know the exact distribution that may exist in real time for the model inputs. By assuming a uniform distribution, we can generate a data set which has information covering the entire input space. When Equation (3) is applied, a data set (Sobol data) containing 442,368 data points is generated. The Sobol data set is used to predict the PM_2.5_ concentration, and the results are then analysed.

Analysis of the inputs using the Sobol method offers insights into the interaction within and between the inputs to predict an output. Sobol analysis provides the sensitivity of individual inputs on the output as a first-order sensitivity (FOS) index and the sensitivity of an input due to its interaction with all of the other inputs as the total-order sensitivity (TOS) index. Both indices range between 0 and 1, with 0 denoting no effect and 1 indicating high effect on the model output variance. FOS equal to TOS indicates that the input does not interact with other inputs, whereas a large difference between FOS and TOS indicates higher-order effects (the input has a strong interaction with the other input parameters). A value of FOS or TOS equal to zero indicates that the input has no impact on the prediction of the model. Sensitivity analysis only identifies the impact of input variability on the model output but not its cause [69].

Categorical variables are not directly supported in the SAlib library. Therefore, the indices of categorical variables were rounded to the closest integer as used in the general probabilistic framework [113]. One thousand RF models that were fit for the baseline model were applied to the Sobol data to predict PM_2.5_ concentrations. Each of the 1000 runs were analysed to produce FOS and TOS. The mean of the FOS and TOS over 1000 model runs was then calculated.

Through GSA, two models were developed, one with predictors with an FOS index greater than 0.01 (GSA_parsimonious) and a second one with the predictors from the GSA_parsimonious and street types (GSA_streets). The RF models were retrained with both GSA models 1000 times each and then validated.

### 2.3. Model Validation and Assessment

#### 2.3.1. Validation

Model validation is an essential part of model development for assessing the general performance and stability of the model. All six models—baseline, RFE, RFECV_baseline, RFECV_parsimonious, GSA_parsimonious, and GSA_streets—were compared to one another to identify the best method for this type of LURM study.

**Hold-out Validation**: Hold-out validation (HOV) is the simplest type of validation of ML models. The data set is divided into a two sets: one for testing and the second for training. The test data set is used to validate the model [79]. The features of the test data set are used to predict PM_2.5_ and are compared against the observed PM_2.5_ concentration. HOV validation may be problematic if the test data set are not representative of the training data set. However, in this work, the range of test data set was similar to that of the training data. Additionally, by means of Monte Carlo runs, the uncertainty of the model could be accounted for.

**BLUME Validation**: The training of the RF models uses AQS data. Since AQS data involve a degree of uncertainty regarding quality, we used data from the official air quality stations run by the city of Berlin for the model validation data (BLUME_val).

BLUME_LURM data were applied to each location, and the six RF models were used to predict the PM_2.5_ concentration. The predicted concentration was compared to the BLUME_val data for assessing and validating the models.

#### 2.3.2. Model Assessment

The assessment of predictive models is a crucial aspect to evaluating the model performance and understanding its strengths and weaknesses. Different regression metrics such as coefficient of determination (R^2^), mean squared error (MSE), root mean squared error (RMSE), scatter index or normalised root mean squared error (NRMSE) [114], and mean absolute error (MAE) were used in this study to evaluate the models. The specific meaning of each of the metrics are detailed in Garreta et al. (2017) [115].

RMSE, MSE, and MAE evaluate the model by examining the absolute errors between the predicted and true or observed values. MSE provides insight into outliers by squaring the difference between true and predicted values. RMSE provides the square root of the MSE, which is easier to interpret as it has the same unit as the predicted variable. RMSE places emphasis on the larger errors. Therefore, it is an important parameter to assess a model that is biased in the upper extremes. This is because there are legally set limit values above which there are penalties due to increased health risk. MAE provides a straightforward understanding of a model’s prediction errors. It calculates the average absolute error magnitude of the model by calculating the mean absolute difference between the true and predicted values [115]. NRMSE calculates the variance between the different models as a function of RMSE and the range of observations [114]. Lower NRMSE, RMSE, MSE, and MAE values indicate less residual variance for a model, thus suggesting a better model, with a value of zero being the best model.

## 3. Results

### 3.1. Feature Selection

**Figure 4:**
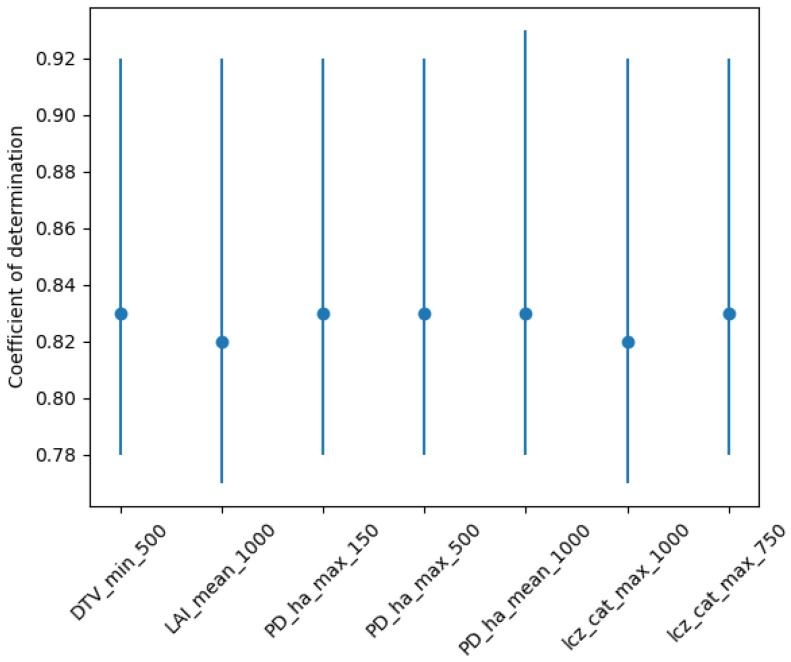
The mean test accuracy of predictors selected using RFE with cross-validation. The *Y*-axis shows the mean test accuracy, and the *X*-axis shows the selected predictors: leaf area index (LAI), local climate zone (LCZ), population density per hectare (PD_ha), and daily traffic volume (DTV). Categorical data are indicated by “cat” after the name of the predictor. The number at the end of the predictor indicates the buffer size in meter and whether the mean, minimum (min), or maximum (max) of the predictor within the buffer is considered.

The RFECV method is used to ascertain those features that are important to develop the model. Monte Carlo runs are carried out, and the features with a mean importance rank of less than two are chosen as the important predictors. Figure 4 shows the coefficient of determination of the model when the model is trained with the particular predictor as its important feature. The point represents the mean R^2^ of 1000 model runs, and the error bars show the spread of the model.

**Figure 5:**
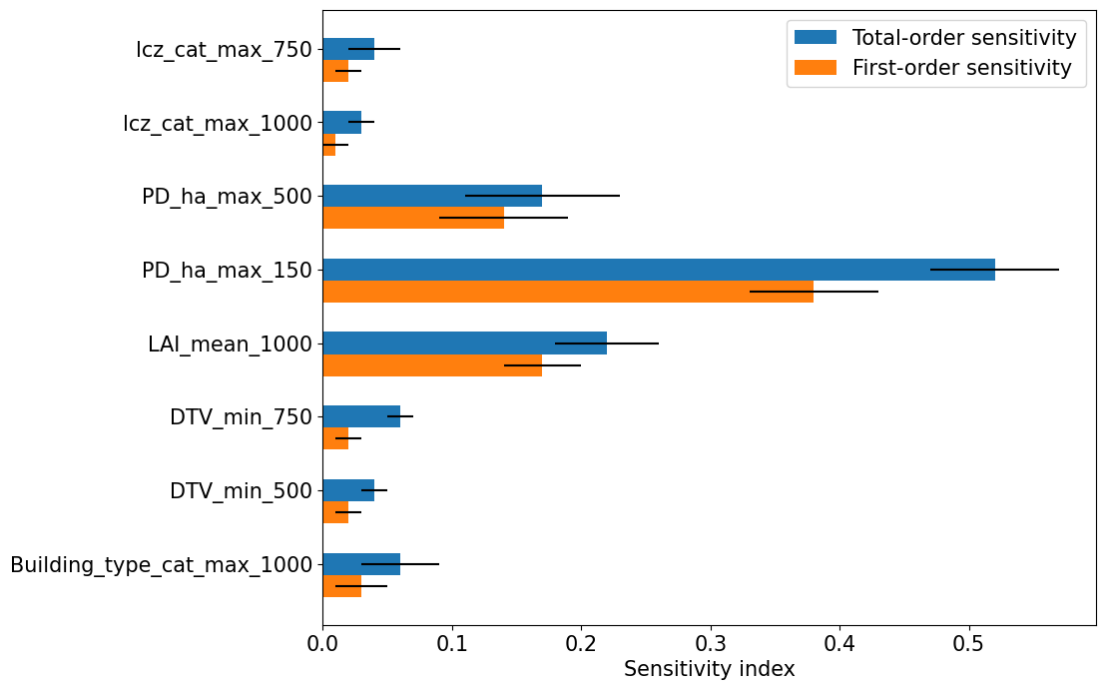
Variance decomposition of the random forest model through attribution of the input to the model output variance using the Sobol method. The *X*-axis shows the sensitivity indices and both the total order sensitivity of the predictors (blue) and the first-order sensitivity (orange) of the predictors. The *Y*-axis shows the predictors used: leaf area index (LAI), local climate zone (LCZ), population density per hectare (PD_ha), daily traffic volume (DTV), and building type. Categorical data are indicated by “cat” after the name of the predictor. The number at the end of the predictor indicates the buffer size in meter and whether the minimum (min) or maximum (max) of the predictor within the buffer is considered.

To select features using GSA, the Sobol method is used. The mean over 1000 runs is calculated, and all inputs with an FOS of greater than 0.01 (see Figure 5) are considered significant for the model. The higher-order effects are indicated by the difference between FOS and TOS. Population density shows a larger influence on the model output followed by LAI and DTV. The error bar (black line) indicates the spread of the sensitivity indices of the predictor over 1000 model runs. The LURM features selected using RFECV as shown in Figure 4 are also selected by GSA (Figure 5).

### 3.2. Model Validation

**Figure 6:**
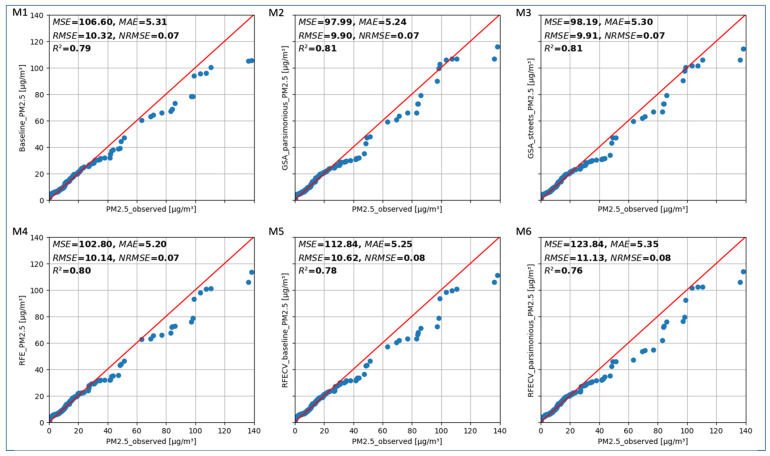
Q-Q plot showing the data distributions and model metrics of six random forest models assessed using hold-out validation. The red line shows the best-fit line and the blue circles show the data points. The top left corner of the Q-Q plots show the MSE, MAE, RMSE, NRMSE, and R^2^ metrics for the baseline (M1), GSA_parsimonious (M2), GSA_streets models (M3), RFE (M4), RFECV_baseline (M5), and RFECV_parsimonious models (M6) respectively.

**Figure 7:**
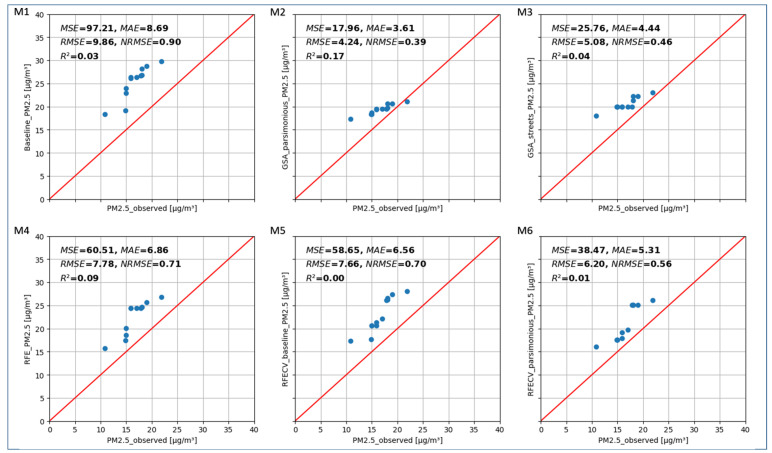
Q-Q plot showing the data distributions and model metrics of six random forest models assessed using BLUME station data for validation. The red line shows the best-fit line and the blue circles show the data points. The top left corner of the Q-Q plots show the metrics MSE, MAE, RMSE, NRMSE and R^2^ for the models baseline (M1), GSA_parsimonious (M2), GSA_streets models (M3), RFE (M4), RFECV_baseline (M5) and RFECV_parsimonious models, (M6) respectively.

Six RF models, baseline (M1), GSA_parsimonious (M2), GSA_streets (M3), RFE (M4), RFECV_baseline (M5), and RFECV_parsimonious (M6), were trained and validated using HOV (Figure 6) and BLUME data (Figure 7). Each of the Q-Q plots in Figure 6 and Figure 7 shows a 45° line in red which indicates the ideal distribution line (1:1) and metrics of the evaluated model. The model metrics show the overall performance of the models.

Figure 6 shows that all six models similarly follow the 1:1 line. The concentrations below 5 µg/m^3^ appear to be overestimated for the GSA_parsimonious (Figure 6 (M2)) and GSA_streets (Figure 6 (M3)) models, while those below 7 µg/m^3^ appear to be overestimated for the baseline (Figure 6 (M1)), RFE (Figure 6 (M4)), RFECV_baseline (Figure 6 (M5)), and RFECV_parsimonious (Figure 6 (M6)) models. For concentrations between 25 µg/m^3^ and 30 µg/m^3^, the baseline (Figure 6 (M1)), RFE (Figure 6 (M4)), and RFECV_baseline (Figure 6 (M6)) models, which contain all the predictors, appear to follow the 1:1 line better as compared to the parsimonious models. For concentrations above 30 µg/m^3^, all the models appear to underestimate the concentration. The model metrics MAE R^2^ and NRMSE show similar results for all the six models, with a slightly better performance being observed for the GSA_parsimonious (Figure 6 (M2)).

Figure 7 shows the data distributions of the BLUME station data and model predictions at the respective BLUME location for all the models. All the models show a bias towards higher values and a low R^2^. The parsimonious models GSA_parsimonious (Figure 7 (M2)), GSA_streets (Figure 7 (M3)), and RFECV_parsimonious (Figure 7 (M6)) have distributions that are closer to the 1:1 line. As opposed to HOV, in BLUME_validation, the GSA_parsimonious model performs best in all the metrics, with an RMSE, MAE, NRMSE, and R^2^ of 4.24 µg/m^3^, 3.61 µg/m^3^, 0.39 and 0.17, respectively, as compared to 9.86 µg/m^3^, 8.69 µg/m^3^, 0.9, and 0.03 in the baseline model, respectively (Figure 7 (M1)).

**Figure 8:**
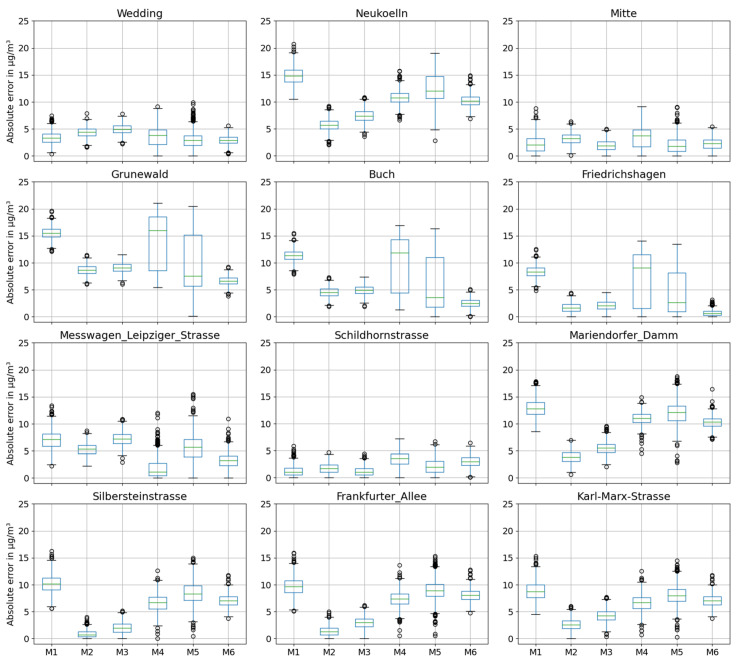
Box plot showing the absolute error at each BLUME station for each of the six models: baseline (M1), GSA_parsimonious (M2), GSA_streets models (M3), RFE (M4), RFECV_baseline (M5), and RFECV_parsimonious (M6). Each plot includes the absolute errors of all the 1000 predicted PM_2.5_ concentrations on the *Y*-axis and the model used on the *X*-axis.

Figure 8 shows the residual plot of the observed BLUME PM_2.5_ concentration and the predicted concentration of the six models: baseline (M1), GSA_parsimonious (M2), GSA_streets models (M3), RFE (M4), RFECV_baseline (M5), and RFECV_parsimonious (M6). All of the models generally appear to overestimate the PM_2.5_ concentration except at two stations, Mitte and Schildhornstrasse, where the concentrations are underestimated. The GSA_parsimonious (M2) is able to predict concentrations with an MAE of less than 10 µg/m^3^ for all the 12 stations and with less than 5 µg/m^3^ at 8 out of 12 locations.

Table 2 shows the root mean square error of each model at the respective BLUME station. At the suburban stations Grunewald, Buch, and Friedrichshagen, the baseline model has an RMSE of 16 µg/m^3^, 11 µg/m^3^, and 8 µg/m^3^, respectively. The RFECV_parsimonious model performs significantly better than does the baseline model, with an RMSE of 7 µg/m^3^, 3 µg/m^3^, and 1 µg/m^3^, respectively. The GSA_parsimonious and GSA_streets models perform second best, with an RMSE of 9 µg/m^3^, 5 µg/m^3^, and 2 µg/m^3^ respectively.

At the urban background stations, all the models perform similarly, with a mean RMSE of 3 µg/m^3^ to 5 µg/m^3^ at Wedding and 2 µg/m^3^ to 4 µg/m^3^ at Mitte. Station Neukoelln, on the other hand, has a mean RMSE between 6 µg/m^3^ and 15 µg/m^3^, with the GSA_parsimonious model performing the best and the baseline model the worst.

The GSA_parsimonious model shows the best performance for traffic stations at Mariendorfer Damm, Silbersteinstrasse, Frankfurter Allee, and Karl-Marx-Strasse, demonstrating significant improvements over the baseline model. On the other hand, for traffic station Schildhornstrasse, all the models show a similar performance, with a mean RMSE between 1 µg/m^3^ and 4 µg/m^3^.

At the traffic station Messwagen-Leipziger-Strasse, the RFE model (RMSE = 3 µg/m^3^) performs significantly better than does the baseline model (RMSE = 7 µg/m^3^). However, the station at Messwagen-Leipziger-Strasse is a mobile station and was therefore not considered. This is the only station where the RFE model performs better compared to the other models.

Including the street predictors improves the predicted PM_2.5_ concentration by approximately 1 µg/m^3^ at the stations Schildhornstrasse and Mitte.

## 4. Discussion

### 4.1. Feature Selection

Figure 4 and Figure 5 show the results of the feature selection process under the RFECV and Sobol methods, respectively. As the features selected using RFECV overlap those chosen by Sobol, the features are discussed together.

The PM_2.5_ concentration depends on local and regional factors (see Figure 5). Four out of the seven inputs selected by RFECV and eight selected by GSA shown in Figure 4 and Figure 5, respectively, are related to population density and daily traffic volume. This is in line with the generally accepted sources of PM_2.5_ concentrations according to the German Environment Agency, wherein approximately 60% of the emissions result from combustion processes, with the largest shares coming from households, small consumer operations such as restaurants, and from road traffic [116,117]. Similar results were found in the study of population density and PM by Borck et al. (2021) [117], who reported that an increase in population density had a direct effect on the increase in PM. The study by Casallas et al. (2024) [118] highlights that the elevated PM levels can be attributed to vehicles and industries, similar to the results found in our study.

LAI is the second most important predictor in both RFECV and GSA model. Since the model pertains to summer months, LAI plays a crucial role. A study on five different urban sites in the United Kingdom by Beckett et al. (2000) [119] indicated that trees of various sizes and ages play a significant role in particulate matter reduction by capturing significant quantities in its foliage. However, the rate of PM uptake can vary between species. A similar study by Nowak et al. (2013) [120] in the United states of America in ten different cities also showed that trees remove fine particles in the air, thereby reducing the urban PM_2.5_ concentration.

LCZs and land-use types are determined by infrastructure planning. It is therefore important to design cities in a way that can effectively reduce the concentration of PM_2.5_ [121]. Building type refers to the age (pre-war, post-war, and current) and usage (residential or office) of the building. Although it is unclear whether the age of buildings or their usage determines the effect on PM_2.5_ concentrations, the building type parameter still has a significant influence on the output of the model.

The GSA also shows that the predictors LAI, LCZ, PD, DTV, and BT have higher-order effects, i.e., a difference between FOS and TOS. This may be because these predictors not only have a direct effect on the predicted PM_2.5_ concentration but can also interact with each other and indirectly affect the predicted PM_2.5_. For example, the type of LCZ has a direct effect on the predicted PM_2.5_ concentrations. However, a change in LCZ, for instance, due to infrastructural developments, can alter PD, DTV, and as BT. Similarly, a change in PD can affect the traffic volume, which in turn affects the LCZ. One such scenario could be when new sealed surfaces (roads) are constructed in barren or green areas to accommodate increased traffic flow. BT, depending on the presence of a residential or commercial type, can affect the local PD, which in turn can affect DTV.

The higher-order effects show the predictors that have the most influence on the model outcome. Domain knowledge is, however, necessary to understand and interpret these effects. This is due to the fact that the sensitivity analysis lacks any information pertaining to the causal directions and cannot differentiate causes from effects. For example, PD and DTV are directly proportional to PM_2.5_ concentration. However, an increase in LAI leads to a decrease in PM_2.5_. Similarly, LCZ is an effect of urban and infrastructure planning and not a cause of it. Therefore, the importance of LCZ as a predictor lies in analysing the underlying infrastructural and land-use plans.

### 4.2. Model Validation

Figure 6 and Figure 7 show the results of the six RF models—baseline (M1), GSA_parsi monious (M2), GSA_streets (M3), RFE (M4), RFECV_baseline (M5), and RFECV_parsimonious (M6)— trained and validated using HOV and BLUME data, respectively.

All six models validated with the HOV data (Figure 6) follow the 1:1 line of the Q-Q plot, showing that the predicted data follow the distribution of the observed data and are comparable. The general bias for concentrations above 30 µg/m^3^ shows that the model cannot be used for modelling extreme cases, as the model would underestimate concentrations in such cases. The model, however, can be used to obtain a general PM_2.5_ spatial distribution, as the annual mean concentration in Berlin is less than 20 µg/m^3^. The parsimonious models using GSA and RFECV capture situations with around 100 µg/m^3^, which only rarely occur if the concentration is measured right next to combustion processes such as vehicles or traffic or smoking. The R^2^ between 0.76 and 0.81 in models M2–M5 as compared to the baseline (M1) (Figure 6) shows that the feature selection process does not reduce the predictive power of the model. The RFE model has the best MAE of 5.20 µg/m^3^ as compared to the other models. A lower MAE indicates a better model since the absolute errors are low. However, since MAE calculates errors linearly, the errors due to outliers are not pronounced. A lower RMSE of a model shows that the model has a lower variance of the residuals. This indicates that the GSA_parsimonious model is able to predict without strong outliers and is therefore better suited for the data set used. RMSE is a deciding factor here since PM_2.5_ has threshold values that when exceeded, can have health risks and legal consequences. Exaggerating such extremes limits the usability of such a model for applications that include information to the general public. The GSA models perform best with a lower MSE/RMSE as compared to the other models. All six models have a low NRMSE of 0.07 to 0.08, indicating that the models perform consistently and robustly to outliers.

Figure 7 shows that the models trained using the AQS data do not generalise well to the BLUME station data. The lower R^2^ in BLUME_val as compared to HOV_val across all models indicates that the model has poor performance when transferred to BLUME locations and that the model does not explain the variance when compared to stationary measurements. The higher NRMSE of 0.38 and low R^2^ of 0.17 of the validation against BLUME station data show that the model is affected by micro-scale structures and therefore cannot be generalised at the regional scale. The sparsity of BLUME stations and the non-availability of AQS measurements at the locations of BLUME stations makes it difficult to make a direct comparison. However, the distribution and R^2^ of the GSA_parsimonious model predictions are better than those of the baseline model, thus indicating that the feature selection using the Sobol method helps to reduce the total uncertainties and improves the explained variance of the model.

Figure 8 shows a direct case-to-case comparison that provides insight into where the model excels and where it falls short. Below, we compare the results of the GSA_parsimonious model (M2) at each BLUME station.

The predicted PM_2.5_ concentration at suburban station Buch is overestimated by 5 µg/m^3^. This is perhaps is due to its location at the northern border of the State of Berlin. Due to its location and the model input parameters being confined to the area of the State of Berlin, the land-use parameters within the buffer zone but outside the State of Berlin are not considered in the model. The predicted concentration at the suburban station Grunewald has the highest mean error at 9 µg/m^3^. The overestimation could be attributed to the station’s location amidst a large green area and away from urbanised areas, which is unlike other suburban stations which are located closer to urbanised areas (see Figure 2). Moreover, the station is located in a dense green area, whereas the training data for the overall model is limited in information about PM_2.5_ concentration in green areas.

The predicted PM_2.5_ concentration at the urban background station Mitte is also underestimated. There have been road works and construction since 2016 in the vicinity of the station Mitte [122,123]. The RF model, however, does not consider road and construction works as a parameter that might explain why the model underestimates the PM_2.5_ concentration at this location. The predicted PM_2.5_ concentration at the urban background station Neukoelln is overestimated by 6 µg/m^3^. The urban background station at Neukoelln is located in a residential area characterised by medium traffic volume. DTV and PD_ha both being significant predictors of the M2 model would explain the overestimation. Neukoelln, along with the stations at Silbersteinstrasse, Karl-Marx-Strasse, and Frankfurter Allee, has some of the highest exceedances of limit values in Germany [124].

The traffic station Mariendorfer Damm is characterized as an area with densely built residential area with a high traffic volume. As with the station at Neukoelln, DTV and PD_ha are both significant predictors of the M2 model, and thus the predicted concentration is overestimated. The traffic station Messwagen-Leipziger-Strasse is a temporary station set up in a measurement car park on the road Leipziger Strasse. The station supposedly did not stay at the same location and was moved around [125] to locations along the street with the highest concentrations. It is therefore difficult to pinpoint the exact location of the station along the street, thereby potentially causing the misrepresentation of the predicted PM_2.5_ concentration.

The station Schildhornstrasse is a traffic station located near a roadside parking area on the highly trafficked street Schildhornstrasse in the neighbourhood of Steglitz. The PM_2.5_ concentration at station Schildhornstrasse is underestimated by the model. The vicinity of Schildhornstrasse is classified as “residential use” in the land-use classification. However, it is also less than 1000 m away from the highways A100 and A103 and from the controversial motorway bridge at Breitenbachplatz, which diverts traffic from the motorway into Schildhornstrasse. The street type classification is disregarded as a significant parameter for the RF model after the sensitivity analysis, as the input parameters are selected based on the mean sensitivity of 1000 model runs. This may result in the contribution of the parameter being overlooked for such a specific case. However, including the street type predictors to the parsimonious model does not have a major effect on the predicted concentration, as seen in Figure 8.

The case-by-case analysis of the predicted PM_2.5_ shows that the GSA_parsimonious model may be able to explain the model metrics at a local scale. However, with a low R^2^ of 0.17, it cannot be used for regional-scale modelling. Other studies have found similar results wherein the LURMs were non-transferable to other areas [29,31,126]. However, the better performance of the GSA_parsimonious model as compared to the baseline model (R^2^ = 0.03) shows that the Sobol GSA helps to improve the model metrics better than the feature elimination or importance techniques offered by the RF algorithms. This goes to show that the higher-order effects of predictors for a LURM play a significant role in the model performance. The ability of GSA to improve models is consistent with studies by Todorov et al. (2023) [76] and Wang et al. (2023) [78].

Overall, the GSA_parsimonious model performs best (Figure 7 M2). This could be because the GSA_parsimonious model has fewer inputs which could reduce the uncertainty propagated through the model. By removing inputs which do not contribute significantly to the model, issues such as unstable coefficients and data overfitting that arise due to multi-collinearity are reduced. Moreover, considering that there are eight predictors in the GSA_parsimonious model as compared to 220 in the baseline model, we can largely reduce the computational time and costs by using the GSA_parsimonious model. RFECV_parsimonious model uses the same features as those of the GSA_parsimonious model, except for DTV_min_750 and building_type_cat_max_1000. The better performance of the GSA_parsimonious model could be due to the larger data set, with 442,368 data points used to perform feature selection as compared to 835 in the RFE and RFECV methods.

## 5. Conclusions

AQSs provide a unique opportunity to collect large quantities of data and capture microscale variations in PM_2.5_ concentrations. Several studies have used AQS data in combination with satellite images and monitoring stations to develop predictive models [42]. In our work, we assessed the potential of using AQS data to develop a LURM incorporating different feature selection methods for a random forest model. The baseline model consisting of all the predictors is subject to feature selection methods RFE, RFECV, and Sobol GSA. RFE and RFECV optimise the model by removing features with the least influence on the model output. While Sobol-GSA, RFE, and RFECV perform variance-based decomposition to highlight the most important features for model prediction, the higher-order effects that can be quantified with Sobol GSA provides an insight into the model dynamics. The developed models were validated using AQS data (hold out validation) and reference station data (BLUME validation). HOV validation showed that the baseline, GSA_parsimonious, GSA_streets, RFE, RFECV_, and RFECV_parsimonious models have similar performances, with the GSA_parsimonious model performing slightly better. BLUME validation showed that the GSA_parsimonious model performs best across all metrics (RMSE = 4.23 µg/m^3^, MSE = 17.93, NRMSE = 0.38, MAE = 3.6 µg/m^3^, and R^2^ = 0.17) as compared to the baseline model, which had significantly poorer metrics (RMSE = 9.86 µg/m^3^, MSE = 97.16, NRMSE = 0.9, MAE = 8.69 µg/m^3^, and R^2^ = 0.03).

The data used for this study included less data from green and blue spaces. Although LCZ in this study included green spaces under the categories of dense trees, scattered trees, low plants, and water, the measurements were carried out in the border of the LCZ and do not represent the LCZ. The GSA_parsimonious model is able to predict PM_2.5_ concentration with an MAE of 9 µg/m^3^ in the suburban station Grunewald and with an MAE of less than or equal to 5 µg/m^3^ in stations Buch and Friedrichshagen. The GSA analysis does not show any impacts of street type classification on the output of the model. Although including the street information reduces the RMSE in two stations, Mitte and Messwagen Leipzigerstrasse, it increases the RMSE in other stations, thereby not providing any significant addition to the GSA process.

The trained GSA model performs well on unseen data from the test sample that uses AQS data, with an MAE of 5.24 µg/m, R^2^ of 0.81, RMSE of 9.9 µg/m^2^, and NRMSE of 0.07. However, the model does not transfer and generalise to data from the reference stations. Therefore, the model can only be used for local analysis and needs to be adapted for regional-scale using data that can capture regional conditions. Other studies have also found problems with transferability using LURM models in general due to differences in training data, data availability, and complex urban structures [29,31].

AQSs can be a good source of training data for predictive models if the sensors are calibrated and if the data are correctly processed. However, the inherent problems with the technology associated with AQSs and the added uncertainty due to a mobile platform makes it a suboptimal choice for regional modelling. Nevertheless, the sensitivity analysis is able to improve the model from an R^2^ of 0.03 in the baseline model to 0.17 in the GSA_parsimonious model, while reducing the number of predictors significantly. The same effect is not as pronounced with the use of RFE and RFECV techniques. This is a proof of concept that using GSA is a highly useful technique for predictor screening when using AQSs.

RF is one of the commonly used ML methods used in regression analysis for air quality. However, with the increasing popularity of ML techniques, other methods such as neural networks [127], coupled deep learning models [78], and stacked ensemble methods [47] show further promising capabilities for predictive modelling.

Feature elimination methods enable the creation of an easier, faster, and parsimonious model, which can reduce the computational intensity and the uncertainty propagated through the model. Although RF has the ability to handle noisy data and has feature importance and elimination techniques available in its algorithm, the GSA Sobol method outperforms these in its feature selection techniques.Potentially, such an analysis can be used to model large study areas with reduced computational intensity and without compromising the predictive capacity.

The higher-order effects show that population density and traffic volume have the largest impact on the outcome of the model. This is in line with other studies suggesting that the combustion processes from households and traffic contribute the most to PM_2.5_ concentrations. These are followed by the leaf area index, local climate zones, and building type come in next, which emphasises the importance of efficient city-planning measures that take PM_2.5_ into consideration, as PM_2.5_ has been reported to have an adverse impact on the cardiovascular and pulmonary health of the population [128,129,130].

The higher-order effects of the sensitivity analysis of our study are valid for local scales and can provide key information for urban planners. With fewer factors to consider, urban planners can make informed decisions to optimise land-use planning. However, sensitivity analysis lacks insight into causal direction and therefore domain knowledge needs to be applied to the higher-order effects. Combining the information of the predictors with the highest sensitivity and domain knowledge on the interactions of these predictors can enhance this study through the inclusion of a causal analysis. This analysis could be performed using packages such as DoWhy [131,132] and provide information that can be used for risk management by means of scenario analysis by policy makers, urban planners, and health officials.

## Figures and Tables

**Table 1:** Summary of bicycle rounds carried out for the study in Berlin-Hermsdorf, Berlin-Adlershof, and Berlin-Charlottenburg-Ernst-Reuter-Platz. For locations see Figure 1.

Location	Measurement Period	Approximate Route Length (km)	Total Number of Measurement Rounds	Number of Rounds Considered for Analysis
Berlin-Hermsdorf	Summer/Winter 2018/2019	18	15	2
Berlin-Charlottenburg	Summer 2018	21	7	5
Berlin-Adlershof	Summer 2018	27	16	16

**Table 2:** Root mean squared error (RMSE) of the predicted PM_2.5_ concentration at each Berlin air quality station. The predictions were carried out using six models, baseline (M1), GSA_parsimonious (M2), GSA_streets (M3), RFE (M4), RFECV_baseline (M5), and RFECV_parsimonious (M6).

Station Type and Location	RMSE_M1 µg/m^3^	RMSE_M2 µg/m^3^	RMSE_M3 µg/m^3^	RMSE_M4 µg/m^3^	RMSE_M5 µg/m^3^	RMSE_M6 µg/m^3^
Urban background						
Wedding	3.55	4.48	5.05	3.96	3.34	3.08
Neukoelln	14.97	5.85	7.52	10.88	12.78	10.32
Mitte	2.46	3.36	2.21	3.79	2.56	2.53
Suburban						
Grunewald	15.52	8.74	9.13	14.49	11.11	6.72
Buch	11.39	4.63	5.01	10.69	7.73	2.66
Friedrichshagen	8.39	1.92	2.27	8.28	5.97	0.89
Traffic						
Messwagen-Leipziger-Strasse	7.29	5.38	7.3	2.55	6.1	3.58
Schildhornstrasse	1.57	1.97	1.45	3.64	2.58	3.17
Mariendorfer-Damm	12.96	4.02	5.63	11.05	12.38	10.33
Silbersteinstrasse	10.33	1.10	2.25	6.79	8.85	7.19
Frankfurter Allee	9.87	1.69	3.17	7.50	9.32	8.18
Karl-Marx-Strasse	9.0	2.82	4.43	6.83	8.43	7.20

## Data Availability

The original data presented in the study are openly available in Zenodo at DOI: 10.5281/zenodo.10076056.

## References

[1] Welsch J., Bömermann H., Nagel H. (2011). Data Sources of the Berlin Pilot Project: The Berlin Environmental Atlas and Social Urban Development Monitoring. UMID.

[2] Franck U., Klimeczek H.J., Kindler A. (2014). Social indicators are predictors of airborne outdoor exposures in Berlin. Ecol. Indic..

[3] World Population Review (2024). Europe Cities by Population 2024.

[4] Koeppen W. (2011). Die Waermezonen der Erde, nach der Dauer der heissen, gemaessigten und kalten Zeit und nach der Wirkung der Waerme auf die organische Welt betrachtet (The thermal zones of the earth according to the duration of hot, moderate and cold periods and to the impact of heat on the organic world). Meteorol. Z..

[5] Langner M., Draheim T., Endlicher W., Endlicher W. (2011). Particulate Matter in the Urban Atmosphere: Concentration, Distribution, Reduction – Results of Studies in the Berlin Metropolitan Area. Perspectives in Urban Ecology.

[6] Kessinger S., Minkos A., Dauert U., Feigenspan S., Hellack B., Moravek A., Richter S., Wichmann-Fiebig M. (2023). Luftqualität 2022.

[7] Querol X., Alastuey A., Ruiz C.R., Artiñano B., Hansson H.C., Harrison R.M., Buringh E., ten Brink H.M., Lutz M., Bruckmann P. (2004). Speciation and origin of PM10 and PM2.5 in selected European cities. Atmos. Environ..

[8] Pirjola L., Niemi J.V., Saarikoski S., Aurela M., Enroth J., Carbone S., Saarnio K., Kuuluvainen H., Kousa A., Rönkkö T. (2017). Physical and chemical characterization of urban winter-time aerosols by mobile measurements in Helsinki, Finland. Atmos. Environ..

[9] Beekmann M., Prévôt A.S.H., Drewnick F., Sciare J., Pandis S.N., van der Denier Gon H.A.C., Crippa M., Freutel F., Poulain L., Ghersi V. (2015). In situ, satellite measurement and model evidence on the dominant regional contribution to fine particulate matter levels in the Paris megacity. Atmos. Chem. Phys..

[10] Kerschbaumer A. (2007). On the Aerosol Budget over Berlin. Ph.D. Thesis.

[11] Pültz J., Banzhaf S., Thürkow M., Kranenburg R., Schaap M. (2023). Source attribution of particulate matter in Berlin. Atmos. Environ..

[12] Borge R., Narros A., Artíñano B., Yagüe C., Gómez-Moreno F.J., de La Paz D., Román-Cascón C., Díaz E., Maqueda G., Sastre M. (2016). Assessment of microscale spatio-temporal variation of air pollution at an urban hotspot in Madrid (Spain) through an extensive field campaign. Atmos. Environ..

[13] Wagener D.K., Selevan S.G., Sexton K. (1995). The importance of human exposure information: A need for exposure-related data bases to protect and promote public health. Annu. Rev. Public Health.

[14] Diener A., Mudu P. (2021). How can vegetation protect us from air pollution? A critical review on green spaces’ mitigation abilities for air-borne particles from a public health perspective—With implications for urban planning. Sci. Total Environ..

[15] Hoek G., Beelen R., de Hoogh K., Vienneau D., Gulliver J., Fischer P., Briggs D. (2008). A review of land-use regression models to assess spatial variation of outdoor air pollution. Atmos. Environ..

[16] Azmi W.N.F.W., Pillai T.R., Latif M.T., Koshy S., Shaharudin R. (2023). Application of land use regression model to assess outdoor air pollution exposure: A review. Environ. Adv..

[17] Ryan P.H., LeMasters G.K. (2007). A review of land-use regression models for characterizing intraurban air pollution exposure. Inhal. Toxicol..

[18] Lin Y.C., Lin Y.T., Chen C.R., Lai C.Y. (2025). Meteorological and traffic effects on air pollutants using Bayesian networks and deep learning. J. Environ. Sci..

[19] Dai H., Huang G., Zeng H. (2023). Multi-objective optimal dispatch strategy for power systems with Spatio-temporal distribution of air pollutants. Sustain. Cities Soc..

[20] Schneider R., Vicedo-Cabrera A.M., Sera F., Masselot P., Stafoggia M., de Hoogh K., Kloog I., Reis S., Vieno M., Gasparrini A. (2020). A Satellite-Based Spatio-Temporal Machine Learning Model to Reconstruct Daily PM2.5 Concentrations across Great Britain. Remote Sens..

[21] Handschuh J., Erbertseder T., Schaap M., Baier F. (2022). Estimating PM2.5 surface concentrations from AOD: A combination of SLSTR and MODIS. Remote Sens. Appl. Soc. Environ..

[22] Galán-Madruga D., Broomandi P., Satyanaga A., Jahanbakhshi A., Bagheri M., Fathian A., Sarvestan R., Cárdenas-Escudero J., Cáceres J.O., Kumar P. (2025). A methodological framework for estimating ambient PM2.5 particulate matter concentrations in the UK. J. Environ. Sci..

[23] Mohammadi F., Teiri H., Hajizadeh Y., Abdolahnejad A., Ebrahimi A. (2024). Prediction of atmospheric PM2.5 level by machine learning techniques in Isfahan, Iran. Sci. Rep..

[24] Maronga B., Banzhaf S., Burmeister C., Esch T., Forkel R., Fröhlich D., Fuka V., Gehrke K.F., Geletič J., Giersch S. (2020). Overview of the PALM model system 6.0. Geosci. Model Dev..

[25] Skamarock W.C., Klemp J.B., Dudhia J., Gill D.O., Liu Z., Berner J., Wang W., Powers J.G., Duda M.G., Barker D.M. (2019). A Description of the Advanced Research WRF Model Version 4.3.

[26] Tanzer R., Malings C., Hauryliuk A., Subramanian R., Presto A.A. (2019). Demonstration of a Low-Cost Multi-Pollutant Network to Quantify Intra-Urban Spatial Variations in Air Pollutant Source Impacts and to Evaluate Environmental Justice. Int. J. Environ. Res. Public Health.

[27] Liao D., Peuquet D.J., Duan Y., Whitsel E.A., Dou J., Smith R.L., Lin H.M., Chen J.C., Heiss G. (2006). GIS approaches for the estimation of residential-level ambient PM concentrations. Environ. Health Perspect..

[28] Briggs D.J., Collins S., Elliot P., Fischer P., Kingham S., Lebret E., Pryl K., van Reeuwijk H., Smallbone K., van der Veen A. (1997). Mapping urban air pollution using GIS: A regression-based approach. Int. J. Geogr. Inf. Sci..

[29] Merbitz H., Fritz S., Schneider C. (2012). Mobile measurements and regression modeling of the spatial particulate matter variability in an urban area. Sci. Total Environ..

[30] Tian Y., deSouza P., Mora S., Yao X., Duarte F., Norford L.K., Lin H., Ratti C. (2022). Evaluating the Meteorological Effects on the Urban Form-Air Quality Relationship Using Mobile Monitoring. Environ. Sci. Technol..

[31] Eeftens M., Beelen R., de Hoogh K., Bellander T., Cesaroni G., Cirach M., Declercq C., Dėdelė A., Dons E., de Nazelle A. (2012). Development of Land Use Regression models for PM_2.5_, PM_2.5_ absorbance, PM_10_ and PM_coarse_ in 20 European study areas; results of the ESCAPE project. Environ. Sci. Technol..

[32] Ge Y., Yang Z., Lin Y., Hopke P.K., Presto A.A., Wang M., Rich D.Q., Zhang J. (2023). Generating High Spatial Resolution Exposure Estimates from Sparse Regulatory Monitoring Data. Atmos. Environ..

[33] Aguilera I., Eeftens M., Meier R., Ducret-Stich R.E., Schindler C., Ineichen A., Phuleria H.C., Probst-Hensch N., Tsai M.Y., Künzli N. (2015). Land use regression models for crustal and traffic-related PM2.5 constituents in four areas of the SAPALDIA study. Environ. Res..

[34] de Hoogh K., Gulliver J., van Donkelaar A., Martin R.V., Marshall J.D., Bechle M.J., Cesaroni G., Pradas M.C., Dedele A., Eeftens M. (2016). Development of West-European PM_2.5_ and NO_2_ land use regression models incorporating satellite-derived and chemical transport modelling data. Environ. Res..

[35] Kumar S., Mishra S., Singh S.K. (2020). A machine learning-based model to estimate PM2.5 concentration levels in Delhi’s atmosphere. Heliyon.

[36] Alfano B., Barretta L., Del Giudice A., de Vito S., Di Francia G., Esposito E., Formisano F., Massera E., Miglietta M.L., Polichetti T. (2020). A Review of Low-Cost Particulate Matter Sensors from the Developers’ Perspectives. Sensors.

[37] Ye Y., Geng P. (2023). A Review of Air Pollution Monitoring Technology for Ports. Appl. Sci..

[38] Hernández-Gordillo A., Ruiz-Correa S., Robledo-Valero V., Hernández-Rosales C., Arriaga S. (2021). Recent advancements in low-cost portable sensors for urban and indoor air quality monitoring. Air Qual. Atmos. Health.

[39] Schneider C., Sauter T., Venkatraman Jagatha J., Chacón-Mateos M., Vogt U. (2023). Sensoren zur Messung von Luftschadstoffen: Möglichkeiten und Grenzen sowie Hinweise zu deren Einsatz.

[40] Munir S., Mayfield M., Coca D., Jubb S.A., Osammor O. (2019). Analysing the performance of low-cost air quality sensors, their drivers, relative benefits and calibration in cities—A case study in Sheffield. Environ. Monit. Assess..

[41] Ngo N.S., Asseko S.V.J., Ebanega M.O., Allo’o Allo’o S.M., Hystad P. (2019). The relationship among PM2.5, traffic emissions, and socioeconomic status: Evidence from Gabon using low-cost, portable air quality monitors. Transp. Res. Part D Transp. Environ..

[42] Oyola P., Carbone S., Timonen H., Torkmahalleh M., Lindén J. (2022). Editorial: Rise of Low-Cost Sensors and Citizen Science in Air Quality Studies. Front. Environ. Sci..

[43] Snyder E.G., Watkins T.H., Solomon P.A., Thoma E.D., Williams R.W., Hagler G.S.W., Shelow D., Hindin D.A., Kilaru V.J., Preuss P.W. (2013). The changing paradigm of air pollution monitoring. Environ. Sci. Technol..

[44] Johnson K.K., Bergin M.H., Russell A.G., Hagler G.S.W. (2018). Field Test of Several Low-Cost Particulate Matter Sensors in High and Low Concentration Urban Environments. Aerosol Air Qual. Res..

[45] Mahajan S., Kumar P. (2020). Evaluation of low-cost sensors for quantitative personal exposure monitoring. Sustain. Cities Soc..

[46] Venkatraman Jagatha J., Klausnitzer A., Chacón-Mateos M., Laquai B., Nieuwkoop E., van der Mark P., Vogt U., Schneider C. (2021). Calibration Method for Particulate Matter Low-Cost Sensors Used in Ambient Air Quality Monitoring and Research. Sensors.

[47] Lim C.C., Kim H., Vilcassim M.J.R., Thurston G.D., Gordon T., Chen L.C., Lee K., Heimbinder M., Kim S.Y. (2019). Mapping urban air quality using mobile sampling with low-cost sensors and machine learning in Seoul, South Korea. Environ. Int..

[48] Milà C., Ballester J., Basagaña X., Nieuwenhuijsen M.J., Tonne C. (2023). Estimating daily air temperature and pollution in Catalonia: A comprehensive spatiotemporal modelling of multiple exposures. Environ. Pollut..

[49] Liu B., Jin Y., Xu D., Wang Y., Li C. (2021). A data calibration method for micro air quality detectors based on a LASSO regression and NARX neural network combined model. Sci. Rep..

[50] Li W., Ma D., Fu J., Qi Y., Shi H., Ni T. (2023). A quantitative exploration of the interactions and synergistic driving mechanisms between factors affecting regional air quality based on deep learning. Atmos. Environ..

[51] Du P., Bai X., Tan K., Xue Z., Samat A., Xia J., Li E., Su H., Liu W. (2020). Advances of Four Machine Learning Methods for Spatial Data Handling: A Review. J. Geovisualization Spat. Anal..

[52] Murugan R., Palanichamy N. Smart City Air Quality Prediction using Machine Learning. Proceedings of the 2021 5th International Conference on Intelligent Computing and Control Systems (ICICCS).

[53] Maaloul K., Brahim L. (2022). Comparative Analysis of Machine Learning for Predicting Air Quality in Smart Cities. WSEAS Trans. Comput..

[54] Danesh Yazdi M., Kuang Z., Dimakopoulou K., Barratt B., Suel E., Amini H., Lyapustin A., Katsouyanni K., Schwartz J. (2020). Predicting Fine Particulate Matter (PM2.5) in the Greater London Area: An Ensemble Approach using Machine Learning Methods. Remote Sens..

[55] Analitis A., Barratt B., Green D., Beddows A., Samoli E., Schwartz J., Katsouyanni K. (2020). Prediction of PM2.5 concentrations at the locations of monitoring sites measuring PM10 and NOx, using generalized additive models and machine learning methods: A case study in London. Atmos. Environ..

[56] Du Y., You S., Liu W., Basang T., Zhang M. (2023). Spatiotemporal evolution characteristics and prediction analysis of urban air quality in China. Sci. Rep..

[57] Tian H., Zhao Y., Luo M., He Q., Han Y., Zeng Z. (2021). Estimating PM_2.5_ from multisource data: A comparison of different machine learning models in the Pearl River Delta of China. Urban Clim..

[58] Mandal S., Madhipatla K.K., Guttikunda S., Kloog I., Prabhakaran D., Schwartz J.D. (2020). Ensemble averaging based assessment of spatiotemporal variations in ambient PM2.5 concentrations over Delhi, India, during 2010–2016. Atmos. Environ..

[59] Chen J., de Hoogh K., Gulliver J., Hoffmann B., Hertel O., Ketzel M., Bauwelinck M., van Donkelaar A., Hvidtfeldt U.A., Katsouyanni K. (2019). A comparison of linear regression, regularization, and machine learning algorithms to develop Europe-wide spatial models of fine particles and nitrogen dioxide. Environ. Int..

[60] Yin P.Y., Tsai C.C., Day R.F. (2019). PSO active learning of XGBoost and spatiotemporal data for PM2.5 sensor calibration. IOP Conf. Ser. Earth Environ. Sci..

[61] Raheja G., Nimo J., Appoh E.K.E., Essien B., Sunu M., Nyante J., Amegah M., Quansah R., Arku R.E., Penn S.L. (2023). Low-Cost Sensor Performance Intercomparison, Correction Factor Development, and 2+ Years of Ambient PM2.5 Monitoring in Accra, Ghana. Environ. Sci. Technol..

[62] Zamani Joharestani M., Cao C., Ni X., Bashir B., Talebiesfandarani S. (2019). PM2.5 Prediction Based on Random Forest, XGBoost, and Deep Learning Using Multisource Remote Sensing Data. Atmosphere.

[63] Schonlau M. (2020). The random forest algorithm for statistical learning. Stata J..

[64] Zhang Z., Hörmann G., Huang J., Fohrer N. (2023). A Random Forest-Based CA-Markov Model to Examine the Dynamics of Land Use/Cover Change Aided with Remote Sensing and GIS. Remote Sens..

[65] Cutler A., Cutler D.R., Stevens J.R., Zhang C. (2012). Random Forests. Ensemble Machine Learning.

[66] Bentéjac C., Csörgő A., Martínez-Muñoz G. (2021). A comparative analysis of gradient boosting algorithms. Artif. Intell. Rev..

[67] Breiman L. (2001). Random Forests. Mach. Learn..

[68] Pedregosa F., Varoquaux G., Gramfort A., Michel V., Thirion B., Grisel O., Blondel M., Prettenhofer P., Weiss R., Dubourg V. (2011). Scikit-learn: Machine Learning in Python. J. Mach. Learn. Res..

[69] Saltelli A., Ratto M., Andres T., Campolongo F., Cariboni J., Gatelli D., Saisana M., Tarantola S. (2008). Global Sensitivity Analysis: The Primer.

[70] Berrocal V.J., Guan Y., Muyskens A., Wang H., Reich B.J., Mulholland J.A., Chang H.H. (2020). A comparison of statistical and machine learning methods for creating national daily maps of ambient PM_2.5_ concentration. Atmos. Environ..

[71] Tang D., Zhan Y., Yang F. (2024). A review of machine learning for modeling air quality: Overlooked but important issues. Atmos. Res..

[72] Guyon I., Weston J., Barnhill S., Vapnik V. (2002). Gene Selection for Cancer Classification using Support Vector Machines. Mach. Learn..

[73] Hastie T., Tibshirani R., Friedman J. (2009). The Elements of Statistical Learning.

[74] Murray-Smith D.J. (2015). Sensitivity Analysis for Model Evaluation. Testing and Validation of Computer Simulation Models: Principles, Methods and Applications.

[75] Hamby D.M. (1994). A review of techniques for parameter sensitivity analysis of environmental models. Environ. Monit. Assess..

[76] Todorov V., Dimov I. (2023). Unveiling the Power of Stochastic Methods: Advancements in Air Pollution Sensitivity Analysis of the Digital Twin. Atmosphere.

[77] Zhang X.Y., Trame M.N., Lesko L.J., Schmidt S. (2015). Sobol Sensitivity Analysis: A Tool to Guide the Development and Evaluation of Systems Pharmacology Models. CPT Pharmacometrics Syst. Pharmacol..

[78] Wang S., Ren Y., Xia B., Liu K., Li H. (2023). Prediction of atmospheric pollutants in urban environment based on coupled deep learning model and sensitivity analysis. Chemosphere.

[79] Mosier C.I.I. (1951). Problems and Designs of Cross-Validation 1. Educ. Psychol. Meas..

[80] Berliner Luftgütemessnetz (2023). Aktueller Luftqualitätsindex|Berliner Luftgüte Messnetz (BLUME)|Luftqualität und Luftgüte in Berlin.

[81] WHO (2021). WHO Global Air Quality Guidelines: Particulate Matter (PM2.5 and PM10), Ozone, Nitrogen Dioxide, Sulfur Dioxide and Carbon Monoxide.

[82] Berlin.de Das offizielle Hauptstadtportal Hermsdorf. https://www.berlin.de/ba-reinickendorf/ueber-den-bezirk/ortsteile/hermsdorf/artikel.84992.php.

[83] Berlin.de Das offizielle Hauptstadtportal Charlottenburg. https://www.berlin.de/special/stadtteile/charlottenburg/.

[84] Berlin.de Das offizielle Hauptstadtportal Adlershof. https://www.berlin.de/special/stadtteile/treptow/915023-5170850-adlershof.html.

[85] Fenner D., Meier F., Bechtel B., Otto M., Scherer D. (2017). Intra and inter ‘local climate zone’ variability of air temperature as observed by crowdsourced citizen weather stations in Berlin, Germany. Meteorol. Z..

[86] Scherer D., Antretter F., Bender S., Cortekar J., Emeis S., Fehrenbach U., Gross G., Halbig G., Hasse J., Maronga B. (2019). Urban Climate Under Change [UC]2—A National Research Programme for Developing a Building-Resolving Atmospheric Model for Entire City Regions. Meteorol. Z..

[87] (2015). Alphasense User Manual OPC-N2 Optical Particle Counter.

[88] (2019). Sensirion Humidity Sensors SHT3x Datasheet.

[89] (2016). Portable Laser Aerosolspectrometer and Dust Monitor Model 1.108/1.109.

[90] Köhler H. (1936). The nucleus in and the growth of Hygroscopic droplets. Trans. Faraday Soc..

[91] Crilley L.R., Singh A., Kramer L.J., Shaw M.D., Alam M.S., Apte J.S., Bloss W.J., Hildebrandt Ruiz L., Fu P., Fu W. (2020). Effect of aerosol composition on the performance of low-cost optical particle counter correction factors. Atmos. Meas. Tech..

[92] Bukowiecki N.P. (2003). Mobile Pollutant Measurement Laboratories—Spatial Distribution and Seasonal Variation of Aerosol Parameters in the Zürich (Switzerland) and Minneapolis (USA) Area. Ph.D. Thesis.

[93] Brantley H.L., Hagler G.S.W., Kimbrough E.S., Williams R.W., Mukerjee S., Neas L.M. (2014). Mobile air monitoring data-processing strategies and effects on spatial air pollution trends. Atmos. Meas. Tech..

[94] Umweltatlas Berlin (2020). Reale Nutzung der bebauten Flächen/Grün- und Freiflächenbestand. https://www.berlin.de/umweltatlas/nutzung/flaechennutzung/.

[95] Umweltatlas Berlin (2014). Straßenverkehr—Emissionen und Immissionen. https://www.berlin.de/umweltatlas/luft/strassenverkehr-emissionen-und-immissionen/2014/zusammenfassung/.

[96] Umweltatlas Berlin (2018). Einwohnerdichte. https://www.berlin.de/umweltatlas/nutzung/einwohnerdichte/2018/zusammenfassung/.

[97] Senatsverwaltung für Mobilität, Verkehr, Klimaschutz und Umwelt (2020). Übergeordnetes Straßennetz von Berlin. https://www.berlin.de/sen/uvk/mobilitaet-und-verkehr/verkehrsplanung/strassen-und-kfz-verkehr/uebergeordnetes-strassennetz/.

[98] Bundesanstalt für Strassenwesen (2017). Fachthemen–Verkehrstechnik-Objektkatalog für das Straßen- und Verkehrswesen (OKSTRA®). https://www.bast.de/DE/Verkehrstechnik/Fachthemen/v2-okstra.html.

[99] Umweltatlas Berlin (2020). Detailnetz-Berlin. https://fbinter.stadt-berlin.de/fb/index.jsp.

[100] Statistik Berlin-Brandenburg (2021). Raumbezüge. www.statistik-berlin-brandenburg.de.

[101] Heldens W., Burmeister C., Kanani-Sühring F., Maronga B., Pavlik D., Sühring M., Zeidler J., Esch T. (2020). Geospatial input data for the PALM model system 6.0: Model requirements, data sources and processing. Geosci. Model Dev..

[102] Liu Y., Wang Y., Zhang J. (2012). New Machine Learning Algorithm: Random Forest. Proceedings of the Third International Conference on Information Computing and Applications.

[103] Berk R.A. (2020). Random Forests. Statistical Learning from a Regression Perspective.

[104] Zhuang H., Wang X., Bendersky M., Najork M. (2020). Feature Transformation for Neural Ranking Models. Proceedings of the 43rd International ACM SIGIR Conference on Research and Development in Information Retrieval.

[105] Barbu A.G., Zhu S.C. (2020). Monte Carlo Methods.

[106] Zhou X., Lin H., Lin H. (2008). Global Sensitivity Analysis. Encyclopedia of GIS.

[107] Sauter T., Venema V. (2011). Natural three-dimensional predictor domains for statistical precipitation downscaling. J. Clim..

[108] Sauter T., Obleitner F. (2015). Assessing the uncertainty of glacier mass-balance simulations in the European Arctic based on variance decomposition. Geosci. Model Dev..

[109] Sobol<sup>′</sup> I.M. (2001). Global sensitivity indices for nonlinear mathematical models and their Monte Carlo estimates. Math. Comput. Simul..

[110] Saltelli A. (2002). Making best use of model evaluations to compute sensitivity indices. Comput. Phys. Commun..

[111] Iwanaga T., Usher W., Herman J. (2022). Toward SALib 2.0: Advancing the accessibility and interpretability of global sensitivity analyses. Socio-Environ. Syst. Model..

[112] Byrne M.D. How many times should a stochastic model be run? An approach based on confidence intervals. Proceedings of the 12th International Conference on Cognitive Modeling, Carleton University.

[113] Baroni G., Tarantola S. (2014). A General Probabilistic Framework for uncertainty and global sensitivity analysis of deterministic models: A hydrological case study. Environ. Model. Softw..

[114] Zambresky L. (1989). A Verification Study of the Global WAM Model December 1987-November 1988.

[115] Garreta R. (2017). Scikit-Learn: Machine Learning Simplified: Implement Scikit-Learn into Every Step of the Data Science Pipeline.

[116] Umweltbundesamt (2022). Emission von Feinstaub der Partikelgröße PM2.5.

[117] Borck R., Schrauth P. (2021). Population density and urban air quality. Reg. Sci. Urban Econ..

[118] Casallas A., Cabrera A., Guevara-Luna M.A., Tompkins A., González Y., Aranda J., Belalcazar L., Mogollon-Sotelo C., Celis N., Lopez-Barrera E. (2024). Air pollution analysis in Northwestern South America: A new Lagrangian framework. Sci. Total Environ..

[119] Paul Beckett K., Freer-Smith P.H., Taylor G. (2000). The capture of particulate pollution by trees at five contrasting urabn sites. Arboric. J..

[120] Nowak D.J., Hirabayashi S., Bodine A., Hoehn R. (2013). Modeled PM2.5 removal by trees in ten U.S. cities and associated health effects. Environ. Pollut..

[121] World Bank (2002). Urban Planning and Air Quality (English): South Asia Urban Air Quality Management Briefing Note.

[122] Grahn R., euroluftbild.de (2019). Berlin aus der Vogelperspektive: Baustelle Bürogebäude des Geschäftshauses JAHO an der Holzmarktstraße am S-Bahnhof Jannowitzbrücke in Berlin, Deutschland. https://www.luftbildsuche.de/info/luftbilder/baustelle-buerogebaeude-geschaeftshauses-jaho-holzmarktstrasse-bahnhof-jannowitzbruecke-berlin-deutschland-419307.html.

[123] Jericho D. (2018). Bürohochhaus statt Waschanlage: An der Jannowitzbrücke wird ein Büropalast mit dem Namen “Jaho” gebaut. Berliner Woche.

[124] (2018). Luftverschmutzung in Berlin: Die Silbersteinstraße hat die höchste Feinstaubbelastung Deutschlands. https://www.tagesspiegel.de/berlin/die-silbersteinstrasse-hat-die-hochste-feinstaubbelastung-deutschlands-4003426.html.

[125] (2023). BLUME-Stationsdaten_088. Stationsdaten: 088 Messwagen Leipziger Str.|Berliner Luftgüte Messnetz (BLUME)|Luftqualität und Luftgüte in Berlin. https://luftdaten.berlin.de/station/mw088#station-info.

[126] Marcon A., de Hoogh K., Gulliver J., Beelen R., Hansell A.L. (2015). Development and transferability of a nitrogen dioxide land use regression model within the Veneto region of Italy. Atmos. Environ..

[127] Liang L., Daniels J., Bailey C., Hu L., Phillips R., South J. (2023). Integrating low-cost sensor monitoring, satellite mapping, and geospatial artificial intelligence for intra-urban air pollution predictions. Environ. Pollut..

[128] Lee B.J., Kim B., Lee K. (2014). Air Pollution Exposure and Cardiovascular Disease. Toxicol. Res..

[129] Pokharel A., Hennessy D.A., Wu F. (2023). Health burden associated with tillage-related PM2.5 pollution in the United States, and mitigation strategies. Sci. Total Environ..

[130] Abed Al Ahad M., Demšar U., Sullivan F., Kulu H. (2023). Long-term exposure to air pollution and mortality in Scotland: A register-based individual-level longitudinal study. Environ. Res..

[131] Sharma A., Kiciman E. (2020). DoWhy: An End-to-End Library for Causal Inference. arXiv.

[132] Blöbaum P., Götz P., Budhathoki K., Mastakouri A.A., Janzing D. (2022). DoWhy-GCM: An extension of DoWhy for causal inference in graphical causal models. arXiv.

